# Tertiary lymphoid structures associate with improved survival in early oral tongue cancer

**DOI:** 10.1186/s12885-022-10208-z

**Published:** 2022-10-30

**Authors:** Alhadi Almangush, Ibrahim O. Bello, Amr Elseragy, Jaana Hagström, Caj Haglund, Luiz Paulo Kowalski, Pentti Nieminen, Ricardo D. Coletta, Antti A. Mäkitie, Tuula Salo, Ilmo Leivo

**Affiliations:** 1 Department of Pathology, University of Helsinki, Haartmaninkatu 3, P.O. Box 21, Helsinki, N00014 Finland; 2 Faculty of Medicine, Research Program in Systems Oncology, University of Helsinki, Helsinki, Finland; 3 Institute of Biomedicine, Pathology, University of Turku, Turku, Finland; 4 Faculty of Dentistry, Misurata University, Misurata, Libya; 5 Department of Oral Medicine and Diagnostic Sciences, King Saud University College of Dentistry, Riyadh, Saudi Arabia; 6 Cancer and Translational Medicine Research Unit, University of Oulu, 90014 Oulu, Finland; 7 Department of Oral and Maxillofacial Diseases, University of Helsinki, Helsinki, Finland; 8 Research Programs Unit, Translational Cancer Medicine, University of Helsinki, Helsinki, Finland; 9 Department of Oral Pathology and Radiology, University of Turku, Turku, Finland; 10 Research Programs Unit, Translational Cancer Medicine, University of Helsinki, P.O. Box 63, 00014 Helsinki, Finland; 11 Department of Surgery, University of Helsinki and Helsinki University Hospital, Helsinki, Finland; 12 Department of Head and Neck Surgery and Otorhinolaryngology, A.C. Camargo Cancer Center, and University of Sao Paulo Medical School, Department of Head and Neck Surgery, São Paulo, SP 05402-000 Brazil; 13 Medical Informatics and Data Analysis Research Group, University of Oulu, Oulu, Finland; 14 Department of Oral Diagnosis and Graduate Program in Oral Biology, School of Dentistry, University of Campinas, Piracicaba, São Paulo, 13414-018 Brazil; 15 Department of Otorhinolaryngology – Head and Neck Surgery, University of Helsinki and Helsinki University Hospital, P.O. Box 263, 00029 Helsinki, Finland; 16 Department of Clinical Sciences, Intervention and Technology, Division of Ear, Nose and Throat Diseases, Karolinska Institutet and Karolinska University Hospital, Stockholm, Sweden; 17 Institute of Biomedicine, Pathology, University of Turku, 20520 Turku, Finland; 18 Turku University Central Hospital, Turku, Finland

**Keywords:** Oral tongue cancer, Early stage, Survival, Tertiary lymphoid structures

## Abstract

**Background:**

The clinical significance of tertiary lymphoid structures (TLSs) is not well-documented in early oral tongue squamous cell carcinoma (OTSCC).

**Methods:**

A total of 310 cases of early (cT1-2N0) OTSCC were included in this multicenter study. Assessment of TLSs was conducted on hematoxylin and eosin-stained sections. TLSs were assessed both in the central part of the tumor and at the invasive front area.

**Results:**

The presence of TLSs associated with improved survival of early OTSCC as presented by Kaplan–Meier survival analyses for disease-specific survival (*P* = 0.01) and overall survival (*P* = 0.006). In multivariable analyses, which included conventional prognostic factors, the absence of TLSs associated with worse disease-specific survival with a hazard ratio (HR) of 1.96 (95% CI 1.09–3.54; *P* = 0.025) and poor overall survival (HR 1.66, 95% CI 1.11–2.48; *P* = 0.014).

**Conclusion:**

Histological evaluation of TLSs predicts survival in early OTSCC. TLSs showed superior prognostic power independent of routine WHO grading and TNM staging system.

## Background

The prognosis of oral tongue squamous cell carcinoma (OTSCC) still remains poor. Therefore, accurate identification of the behavior of each individual OTSCC would serve as the foundation of a successful individualized treatment strategy. In daily practice, however, treatment planning is mostly based on TNM classification, which has a limited accuracy of prediction since within the same stage there may be tumors with different clinical behavior. In addition, a single prognostic parameter is not sufficient for a proper prediction of prognosis, and therefore multiple prognostic factors are necessary and carry more potential than a treatment decision based on a single prognostic criterion [1]. Furthermore, histological prognostic markers that are currently reported in pathology reports do not include parameter/s to assess the host immune response. Therefore, additional prognostic markers are necessary to provide a more specific understanding of tumor behavior in individual cases seen from different points of view, including an immunological aspect. Thus, understanding the interaction between invading cancer cell/s and host immune cells/structures can aid in assessing the clinical behavior of individual tumors.

The local immune response in the tumor microenvironment (TME) has received major research attention in the field of tumor immunology [2]. Tertiary lymphoid structures (TLSs) are defined as cumulative areas (or aggregates) of ectopic lymphocytes that occur in nonlymphoid tissues during inflammation and carcinogenesis [3]. TLSs have been observed in the TME and found to have a pivotal role in the antitumor immune response, and to associate with improved survival in many tumors [3–8]. Histologically, TLSs present as organ-like structures of lymphocytes that can be assessed simply using hematoxylin and eosin (HE) stained slides or using immunohistochemistry [9]. The clinical significance of TLSs has been widely studied recently and has been associated with the response to cancer immunotherapy [10, 11]. In early-stage OTSCC, however, the clinical relevance of TLSs still requires further investigation. To the best of our knowledge, this is the first multi-institutional study to analyze TLSs in a large cohort of early-stage OTSCC.

## Methods

In this study, we included a total of 310 cases who were treated for early OTSCC in the period between 1979 and 2009 at five Finnish university hospitals (Helsinki, Turku, Tampere, Oulu, Kuopio) or at the A.C. Camargo Cancer Center, São Paulo, Brazil, and were previously included in our recent study [12]. The study was conducted with the permission of the above hospitals, the National Supervisory Authority for Welfare and Health in Finland, and the Brazilian Human Research Ethics Committee. We included an unselected series of cases of early-stage oral tongue cancer that were treated primarily by surgery at the participating centers. In all cases, the resection slides stained with hematoxylin and eosin were available for evaluation. We excluded cases that were treated for other head and neck tumors and cases where there were not enough histologic slides for evaluation. We also excluded cases without sufficient follow-up data for survival analyses.

**Fig. 1:**
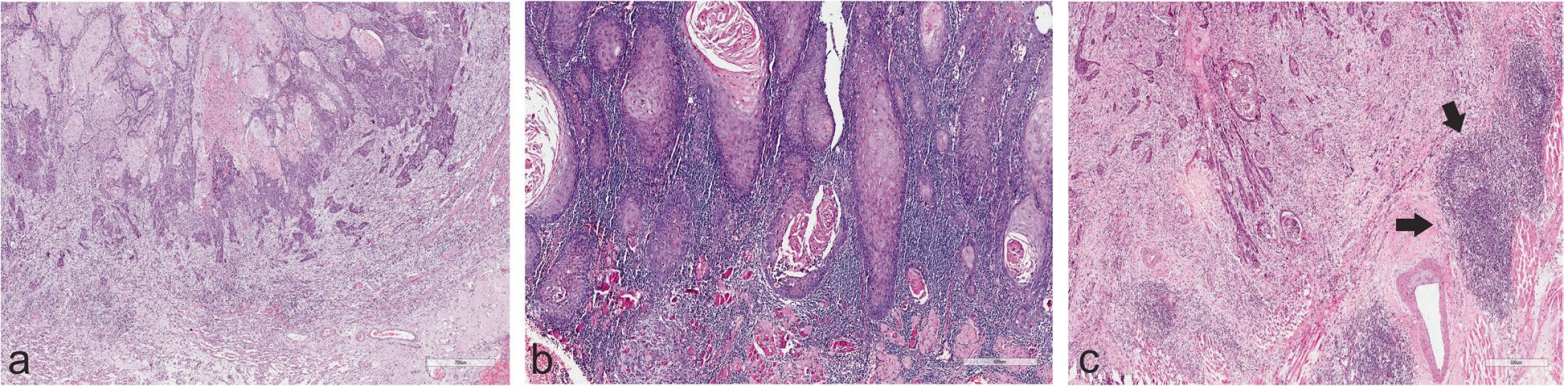
Invasive front and peritumoral areas in early oral tongue squamous cell carcinoma. **A** No observable tertiary lymphoid structures (TLSs). **B** Well-defined lymphoid follicles which can be likened to primary follicles. **C** Two peritumoral secondary lymphoid follicles at the invasive front of a tumor that is otherwise devoid of strong lymphocytic response. One of the aggregates with a germinal center is indicated with arrows with a second smaller one close by

Two researchers (AA, IOB), who were blinded to patient data, assessed TLSs in the HE-stained whole-tissue sections (Fig. 1). We assessed TLSs in the stroma of the body of the tumor and in the stroma at the invasive front area. Samples were classified as:

- i) No TLSs: No lymphoid structures were found in the sample area.
- ii) Lymphoid aggregate/s: Vague, ill-defined clusters of lymphocytes.
- iii) Primary follicle/s: Rounded clusters of lymphocytes without formation of germinal centers.
- iv) Secondary follicle/s: Follicles with germinal center formation.

### Statistical analysis

We used IBM SPSS Statistics (version 25.0) and MedCalc (version 20) for statistical analyses. Univariable and multivariable Cox regression analyses (with reporting of hazard ratios (HR) and 95% confidence interval (95% CI)) were used to assess the relationship between prognostic variables (including TLSs) and survival. Kaplan–Meier curves were also estimated for disease-specific and overall survival analysis. We used the log-rank test to evaluate the statistical significance between the survival curves of the TLSs groups. Disease-specific survival was defined as the time from the date of diagnosis to the date of death from OTSCC or to the time of last follow-up. Overall survival was defined as the time from diagnosis to the date of death due to any cause, or to the time of last follow-up. We categorized the tumors into four groups (No TLSs; Lymphoid aggregates; Primary follicles; Secondary follicle/s) as mentioned above. Further, we divided the samples into two groups based on the presence or absence of TLSs.

### Results

The patients included 164 (52.9%) men and 146 (47.1%) women. The median follow-up time was 57 months, and the median age at the time of diagnosis was 62 years. At the end of follow-up, 63 (20.3%) patients had died of OTSCC, 95 (30.6%) patients were dead of other causes, and 152 (49.0%) patients were alive. With regard to histologic grading, 105 (33.9%) tumors were well differentiated, 130 (41.9%) were moderately differentiated and 75 (24.2%) were poorly differentiated. There were 123 (39.7%) cases classified as T1N0M0 and 187 (60.3%) were T2N0M0.

**Fig. 2:**
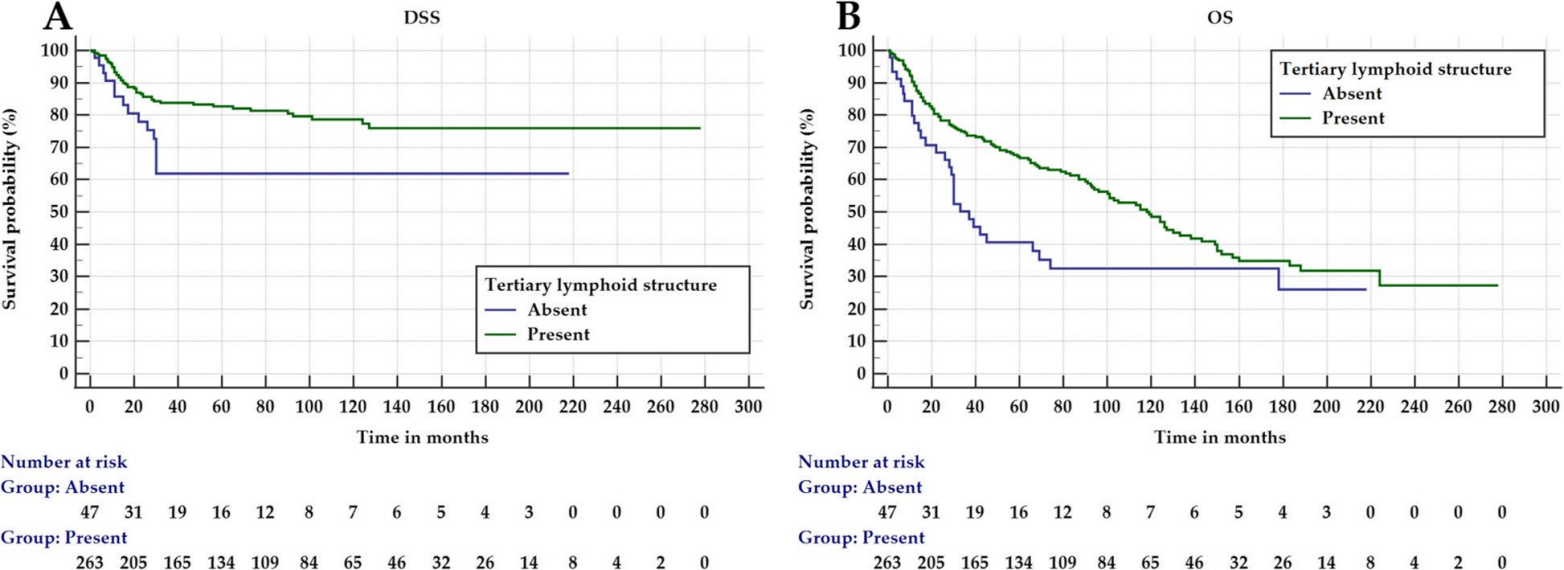
Kaplan–Meier survival curves for early oral tongue cancer patients as classified by the presence of tertiary lymphoid structures (TLSs). Tumors that associate with the presence of TLSs associate significantly with a higher rate of survival. **A** Disease-specific survival (*P* = 0.01). **B** Overall survival (*P* = 0.006)

A total of 263 (84.8%) tumors presented with TLSs in the peritumoral area (i.e. invasive front area), while 47 (15.2%) had no TLSs in this area. In the univariate analyses, cases with no TLSs were associated with a worse disease-specific survival with HR 2.11 (95% CI 1.18–3.78; *P* = 0.012) and a worse overall survival with HR 1.73 (95% CI 1.16–2.57; *P* = 0.007). This was confirmed in multivariable analyses for both disease-specific survival (HR 1.96, 95% CI 1.09–3.54; *P* = 0.025) and overall survival (HR 1.66, 95% CI 1.11–2.48; *P* = 0.014). Kaplan–Meier curves (Fig. 2 A and B) showed a significantly better disease-specific survival (*P* = 0.01) and overall survival (*P* = 0.006) in cases with TLSs in the peritumoral area compared with cases that did not present with any TLSs. On the other hand, TLSs were seen in the stroma of the body of the tumor in only 33.9% of the tumors and these did not associate with survival (*P* > 0.05).

When the cases of this study were reclassified according to the 8th edition of TNM AJCC, 89 (30.7%) of them were T1N0M0 and 201 (69.3%) were T2N0M0. TLSs in the invasive front area associated again with disease-specific survival in both univariate analysis (HR 2.03, 95% CI 1.07–3.85; *P* = 0.03) and multivariate analysis (HR 2.04, 95% CI 1.07–3.91; *P* = 0.031). Similarly, in cases classified according to the 8th edition of AJCC, TLSs associated with overall survival in both univariate analysis (HR 1.78, 95% CI 1.16–2.72; *P* = 0.008) and multivariate analysis (HR 1.87, 95% CI 1.12–2.88; *P* = 0.005). As presented in Table 1, the routine clinicopathologic prognostic parameters including WHO histologic grade, TNM stage (either 7th AJCC or 8th AJCC), and perineural invasion did not associate significantly with survival. Results of the multivariate analysis (Table 1) with all these parameters did not influence the significance of TLSs, indicating independent prognostic nature of TLSs.

**Table 1:** Disease-specific survival and overall survival analyses in a series of 310 patients with early oral tongue squamous cell cancer

**Parameter**	**Number (%)**	**Disease-specific survival**		**Overall survival**	
		**Univariable analysis**	**Multivariable analysis**	**Univariable analysis**	**Multivariable analysis**
		HR (95% CI) *P value*	HR (95% CI) *P* value	HR (95% CI) *P value*	HR (95% CI) *P value*
**Age**		***P* = 0.02**	***P* < 0.02**	***P* < 0.001**	***P* < 0.001**
≤ 60	129 (41.6%)	Reference	Reference	Reference	Reference
> 60	181 (58.4%)	1.89 (1.11–3.21)	1.94 (1.12–3.38)	2.17 (1.55–3.03)	2.32 (1.64–3.29)
**Gender**		*P* = 0.46	*P* = 0.59	*P* = 0.13	***P* = 0.015**
Men	164 (52.9%)	Reference	Reference	Reference	Reference
Women	146 (47.1%)	1.20 (0.73–1.97)	1.15 (0.69–1.93)	0.79 (0.57–1.08)	0.66 (0.47–0.92)
**TNM AJCC 7**		*P* = 0.16	*P* = 0.19	*P* = 0.18	*P* = 0.42
T1N0M0	123 (39.7%)	Reference	Reference	Reference	Reference
T2N0M0	187 (60.3%)	1.47 (0.86–2.51)	1.45 (0.83–2.52)	1.26 (0.90–1.76)	1.15 (0.82–1.63)
**TNM AJCC 8**		*P* = 0.30	*P* = 0.29	*P* = 0.43	*P* = 0.98
T1N0M0	89 (30.7%)	Reference	Reference	Reference	Reference
T2N0M0	201 (69.3%)	1.39 (0.75–2.59)	1.41 (0.74–2.69)	1.17 (0.79–1.70)	1.00 (0.68–1.49)
**WHO Grade**		*P* = 0.63	*P* = 0.51	*P* = 0.71	*P* = 0.94
Grade I & II	235 (75.8%)	Reference	Reference	Reference	Reference
Grade III	75 (24.2%)	1.15 (0.65–2.03)	1.22 (0.68–2.20)	0.93 (0.64–1.35)	0.98 (0.67–1.45)
**Perineural invasion**		*P* = 0.42	*P* = 0.68	*P* = 0.20	*P* = 0.33
No	269 (86.8%)	Reference	Reference	Reference	Reference
Yes	41 (13.2%)	1.32 (0.67–2.59)	1.16 (0.58–2.29)	1.32 (0.86–2.01)	1.24 (0.81–1.90)
**Tertiary lymphoid structure**		***P* = 0.012**	***P* = 0.025**	***P* = 0.007**	***P* = 0.014**
Present	263 (84.8%)	Reference	Reference	Reference	Reference
Absent	47 (15.2%)	2.11 (1.18–3.78)	1.96 (1.09–3.54)	1.73 (1.16–2.57)	1.66 (1.11–2.48)

## Discussion

Immune-related prognostic markers can aid in the clinical assessment of the antitumor immune response and in estimating patient survival. Therefore, such markers have received research attention in the era of cancer immunotherapy and personalized treatment approaches. However, such markers are not presently used in daily practice to assess the immune response of OTSCC. In this multi-institutional study, we assessed tertiary lymphoid structures (TLSs) in HE-stained slides and reported their prognostic significance in early OTSCC.

During invasion, cancer cells can evade immune destruction, but immune cells can still identify and attack cancer cells [13]. The formation of TLSs has similarities with the formation of secondary lymphoid organs [14]. It is speculated that TLSs develop as a result of a prolonged exposure to inflammatory signals [9]. It is well known that tumor-promoting inflammation is one of the hallmarks of cancer [13]. Furthermore, accumulated evidence suggests that TLSs have a role in controlling invasion and metastasis [3, 9], another hallmark of cancer. This might be one of the explanations for the correlation of a good prognosis in many tumor types with the presence of TLSs. This includes lung cancer [4], endometrial cancer [5], gastric cancer [6], breast cancer [15], liver cancer [16] and head and neck cancer [17]. In our current study of early OTSCC, the prognostic impact of TLSs was independent of TNM stage and WHO grade (Table 1). In addition, neither TNM stage nor WHO grade was associated significantly with survival.

Sites of lymphoid neogenesis expressing TLSs have been suggested to have a role in the recruitment of infiltrating lymphocytes [3, 18]. The composition of TLSs includes B cells, T cells, dendritic cells, plasma cells, macrophages, neutrophils, and high endothelial venules [9]. As an assembly of immune cells, TLSs are important sites for the activation of T and B cells to initiate and maintain immune responses against cancer cells [3, 19]. Of note, a recent study by Helmink et al. found that TLSs can promote the response to immune-checkpoint inhibition [10]. In addition, Cabrita et al. reported that TLSs improve survival and response to immunotherapy in melanoma [11]. Such findings support the speculated role of TLSs in an adaptive anticancer immune response, which however, is not yet well-understood [9].

A digital assessment of TLSs in HE-stained slides has been reported with promising value in recent studies on lung cancer [20, 21]. Such a method of assessment can aid in more standardized evaluation of TLSs and in reducing inter-observer variability. Remarkably, it is important to keep in mind the recommendation of WHO classification on breast cancer advising that TLSs should not be counted when assessing stromal tumor-infiltrating lymphocytes [22]. This needs to be considered also in other tumor locations including OTSCC until a better understanding of TLSs and a validation of their prognostic performance in multiple studies can be achieved. In the current study we found a superior prognostic power for TLSs when compared with routinely used prognostic parameters including the TNM stage (both 7th edition and 8th edition), perineural invasion and the WHO grading (Table 1). Due to lack of information about margin status in some cases in this multicenter study, we were not able to compare TLSs with margin status. This shortcoming needs to be addressed in future research.

## Conclusions

TLSs are associated with improved survival in early OTSCC, indicating an association with effective antitumor immunity. Our analysis showed that the absence of TLSs is significantly associated with high mortality. In the future, inducing the formation of TLSs may be one of the strategies for improving patient survival in OTSCC. Meanwhile, TLSs can aid in recognizing patient-to-patient variability with regard to immune status and survival in early OTSCC. Further research is necessary to validate the findings of the current study and to clarify the mechanisms behind the role of TLSs in the antitumor immune response in early OTSCC.

## Data Availability

Data used in this study is available from the corresponding author upon a reasonable request.

## Abbreviations

CI: Confidence interval
HR: Hazard ratio
HE: Hematoxylin and eosin
WHO: World Health Organization
OTSCC: Oral tongue squamous cell carcinoma
TLSs: Tertiary lymphoid structures
